# A prospective observational cohort study to identify inflammatory biomarkers for the diagnosis and prognosis of patients with sepsis

**DOI:** 10.1186/s40560-022-00602-x

**Published:** 2022-03-09

**Authors:** Valentino D’Onofrio, Dries Heylen, Murih Pusparum, Inge Grondman, Johan Vanwalleghem, Agnes Meersman, Reinoud Cartuyvels, Peter Messiaen, Leo A. B. Joosten, Mihai G. Netea, Dirk Valkenborg, Gökhan Ertaylan, Inge C. Gyssens

**Affiliations:** 1 Faculty of Medicine and Life Sciences, Hasselt University, Martelarenlaan 42, 3500 Hasselt, Belgium; 2 Department of Infectious Diseases and Immunity, Jessa Hospital, Hasselt, Belgium; 3 Department of Internal Medicine and Radboud Center for Infectious Diseases, Radboud University Medical Center, Geert Grooteplein Zuid 10, 6525 GA Nijmegen, The Netherlands; 4 Unit Health, Flemish Institute for Technological Research (VITO), Mol, Belgium; 5 Data Science Institute, Hasselt University, Hasselt, Belgium; 6 Department of Nephrology, Jessa Hospital, Hasselt, Belgium; 7 Emergency Department, Jessa Hospital, Hasselt, Belgium; 8 Department of Clinical Biology, Jessa Hospital, Hasselt, Belgium; 9 Human Genomics Laboratory, Craiova University of Medicine and Pharmacy, Craiova, Romania

**Keywords:** Biomarkers, Sepsis, Inflammation, Disease severity

## Abstract

**Background:**

Sepsis is a life-threatening organ dysfunction. A fast diagnosis is crucial for patient management. Proteins that are synthesized during the inflammatory response can be used as biomarkers, helping in a rapid clinical assessment or an early diagnosis of infection. The aim of this study was to identify biomarkers of inflammation for the diagnosis and prognosis of infection in patients with suspected sepsis.

**Methods:**

In total 406 episodes were included in a prospective cohort study. Plasma was collected from all patients with suspected sepsis, for whom blood cultures were drawn, in the emergency department (ED), the department of infectious diseases, or the haemodialysis unit on the first day of a new episode. Samples were analysed using a 92-plex proteomic panel based on a proximity extension assay with oligonucleotide-labelled antibody probe pairs (OLink, Uppsala, Sweden). Supervised and unsupervised differential expression analyses and pathway enrichment analyses were performed to search for inflammatory proteins that were different between patients with viral or bacterial sepsis and between patients with worse or less severe outcome.

**Results:**

Supervised differential expression analysis revealed 21 proteins that were significantly lower in circulation of patients with viral infections compared to patients with bacterial infections. More strongly, higher expression levels were observed for 38 proteins in patients with high SOFA scores (> 4), and for 21 proteins in patients with worse outcome. These proteins are mostly involved in pathways known to be activated early in the inflammatory response. Unsupervised, hierarchical clustering confirmed that inflammatory response was more strongly related to disease severity than to aetiology.

**Conclusion:**

Several differentially expressed inflammatory proteins were identified that could be used as biomarkers for sepsis. These proteins are mostly related to disease severity. Within the setting of an emergency department, they could be used for outcome prediction, patient monitoring, and directing diagnostics.

*Trail registration number:* clinicaltrial.gov identifier NCT03841162.

**Supplementary Information:**

The online version contains supplementary material available at 10.1186/s40560-022-00602-x.

## Introduction

Sepsis is a life-threatening organ dysfunction caused by a dysregulated host response to infection [1, 2]. Septic shock is a subset of sepsis in which profound circulatory, cellular, and metabolic abnormalities are associated with a greater risk of mortality than with sepsis alone [1]. In patients with sepsis, the fast initiation of antibiotic therapy is crucial and each hour delay results in increased risk of mortality [3]. The causative event is an invading pathogen, most frequently *Escherichia coli*, *Staphylococcus aureus*, *Streptococcus pneumoniae*, and *Klebsiella species,* but also influenza virus. Pneumonia, urinary tract infections, and intra-abdominal infections commonly result in sepsis, although the initial site of infection is not always known, e.g., in primary bloodstream infections (BSI) [2, 4]. However, infection sites are related to different causative pathogens and require different antimicrobial treatment.

Organ dysfunction resulting from infection is represented in the new definition of sepsis and defined by an increased sequential organ failure assessment (SOFA) score and is often caused by the dysregulated host response. An increase in SOFA score of 2 or more is associated with an in-hospital mortality of 10% [1]. The host response can be heterogeneous and is characterized not only by excessive inflammation, but also by immune suppression. Ultimately, the host response can be unbalanced and harmful leading to failure to return to homeostasis [5]. The increasing knowledge on host response in sepsis allows for the measurement of inflammation to identify blood biomarkers for a better diagnosis and prognosis of patients with various infections. Several biomarkers for inflammation and infection exist, although they mostly provide an indication on the clinical state of the patient while lacking sensitivity or specificity needed to be used as a diagnostic tool [6]. Two widely used markers are white blood cell count (WBC) and C-reactive protein (CRP) although they lack specificity [7, 8]. In addition, procalcitonin (PCT), a pro-inflammatory biomarker released by monocytes and macrophages, correlates with inflammation intensity and highly increased concentrations are seen in bacterial infections [7–9]. However, a multi-marker approach seems more reliable [6, 8]. Previous studies already reported sensitivity and specificity for prognosis in the intensive care unit (ICU) of 94–96% and 56–94% [10, 11]. Some studies even reported models that perform with better accuracy than PCT [12].Contrarily, other studies reported that circulating biomarkers discriminated poorly between sepsis and non-septic inflammatory reactions [13]. Biomarkers can help in providing a rapid clinical assessment of patients and predict disease severity. Therefore, they could improve outcomes by rapidly guiding triage and the start of adequate treatment [9, 14]. A faster diagnosis of the infection and the likely causative pathogen, will lead to a faster start of targeted antimicrobial therapy, thereby reducing selective pressure for antimicrobial resistance (AMR). A faster prognostic assessment will guide closer patient monitoring. The aim of this study was to identify biomarkers of inflammation for determining the aetiology of the infection and its prognosis in patients with suspected sepsis.

## Materials and methods

### Study design and study patients

This study was part of a prospective observational cohort study performed between February 2019 and April 2020 at a 981-bed teaching hospital (clinicaltrial.gov identifier NCT03841162).

Adult patients presenting with suspected sepsis at the emergency department (ED), the department of infectious diseases/nephrology, or the haemodialysis unit could participate in the study. A suspected sepsis protocol is in place at the hospital, and patient identification is mainly based on physician experience. Therefore, all patients from whom blood cultures were drawn were considered to have suspected sepsis and included in the study. Patients were included after collection of the first set of blood cultures. The blood culture draw, which means the suspicion of sepsis, was the start of the septic episode and the moment of inclusion. All other events happened later.

All patients were followed during hospitalization, and if they developed a new suspected sepsis episode, they could be included multiple times. A new episode was defined as a minimal interval of 7 days between positive cultures with the same pathogen or at least 24 h between positive cultures with different organisms from the same site.

This is a sub-study in which patients with primary BSI, pneumonia, influenza, urosepsis, or other secondary BSI and a SOFA score of 1 or more, to include both patients with and without sepsis, were selected for biomarker identification between February 2019 and March 2020. An analysis on risk factors for patient outcomes of the complete study cohort (1690 episodes of suspected sepsis) was performed earlier [15]. The methods of patient inclusion, microbiological diagnostics, and definitions of infection diagnoses were identical in both analyses.

### Sample collection

Blood cultures, EDTA samples for complete blood count (CDC) and serum and heparin plasma samples for basic laboratory parameters assessing organ function were obtained for each patient at the start of each new episode. Two 9 mL EDTA blood samples were drawn from the same venepuncture. EDTA samples were stored at 4 °C until written informed consent was obtained for a maximum of 3 days. Samples were transferred to the University Biobank Limburg (UBiLim) and centrifuged at 400 g for 10 min. Plasma was separated and centrifuged at 1500 g for 10 min. Four aliquots of 500μL plasma were stored at UBiLim [16] for each patient at − 80 °C until further analysis.

### Microbiological diagnostics

Blood cultures were performed for all patient episodes using the BACTEC FX (Becton Dickinson) system. Bacterial identification was done by MALDI–TOF Biotyper (Bruker). Susceptibility testing was done by the Phoenix system TM 100 (Becton Dickinson). Blood cultures were processed 24 h/day, 7d/week. Other microbiological diagnostics were performed if deemed relevant by the treating physician. This included cultures of urine, lower respiratory tract and samples of specific foci, urinary antigen tests for *Streptococcus pneumoniae* and *Legionella pneumophila,* and PCR for respiratory pathogens on nasopharyngeal swabs.

### Data collection

Relevant data was extracted from patients’ electronic medical files. SOFA-score at the start of a new episode was calculated after blood culture draw for all patients [1, 17]. Recorded patient outcomes were in-hospital mortality, ICU admission at any time during hospital admission, hospital and ICU length of stay (LOS), and the presence of bacteraemia. A composite parameter for worse outcome was made, since mortality was expected to be low, and patients admitted to the ICU are expected to have a more severe disease. Patients who were admitted to the ICU or who died during hospitalization were classified in the worse outcome group.

### Definitions

The final diagnosis of infection was extracted from the treating physicians’ discharge letter and structured and validated according to infectious diseases definitions [1, 18–21] by a researcher (V.D.) and an experienced ID physician (I.C.G.) not involved in the care or consultations of study patients. Sepsis-3 definitions were used to define the presence of sepsis, i.e., all patients with a SOFA score of ≥ 2, and septic shock at the start of an episode [1]. Severe disease was defined as SOFA score of > 4 and compared with less severe disease (SOFA < 2). No distinction was made between community acquired infections and healthcare associated infections. Positive blood cultures were classified as true bacteraemia or as contamination according to CDC guidelines [18]. Positive blood cultures with skin flora, such as coagulase-negative staphylococci, were considered as contaminated when less than two blood cultures bottles from one patient were positive for skin flora. Patients with a positive blood culture with these organisms and a clinical suspicion of an infection of central venous catheters or surgically implanted prosthetic material were considered to have true bacteraemia. Primary BSI was defined as true bacteraemia, without a focus of infection. Patients with a central-line associated BSI (CLABSI) or with confirmed endocarditis were included in this group. Pneumonia was defined as an acute symptomatic infection of the lower respiratory tract, whereby a new infiltrate is demonstrated [19]. Influenza was defined by a positive influenza PCR test. Patients with both influenza and pneumonia were classified as having influenza and were further classified as influenza with or without pneumonia based on the presence of chest X-ray abnormalities. Other viral causes of pneumonia were not detected in this population. Therefore, pneumonia without the presence of influenza was classified as bacterial pneumonia. Secondary bacteraemia patients had true bacteraemia with a urinary tract focus [20], an intra-abdominal focus or a skin and skin structure infection.

### Protein identification

EDTA plasma from patients was analysed using a proteomic multiplex assay (Olink, Uppsala, Sweden), which is a proximity extension assay with oligonucleotide-labelled antibody probe pairs [22]. Samples were analysed with the inflammation panel, which enables analysis of 92 inflammation-related proteins. Briefly, a pair of oligonucleotide-conjugated antibodies to each protein is added to 50μL EDTA plasma. Both antibodies are in close proximity when an antibody–protein–antibody sandwich is formed. This allows hybridization of the oligonucleotides, and an extension reaction forms a unique sequence. The sequences are quantified by qPCR.

### Statistical analyses

Descriptive statistics were used to analyse patient’s characteristics. Continuous data are shown as median (interquartile range (IQR)). Categorical data are reported as number and proportion. A *p* value of < 0.05 was considered statistically significant. Data on protein levels were analysed as normalised protein expression (NPX on a log2 scale). Normalization across batches was performed using bridging samples according to manufacturer’s instructions. Supervised analyses using Welch’s t test were done to search for differences according to aetiology (influenza vs. bacterial infection), severity (SOFA score > 4 versus SOFA score < 2) and outcome (worse versus less severe outcome). This was followed by unsupervised hierarchical clustering and pathway enrichment analyses. Elastic net regression was performed to search for the best biomarker predictors of different outcomes, which was defined as the model with the least number of biomarkers while maintaining an accuracy of ≥ 75%. All analyses were performed using R. Statistical methods are detailed in the Additional file 1.

## Results

### Patient characteristics

In total, 406 episodes of suspected sepsis in 397 patients were included. Table 1 shows the characteristics of the cohort. The median age was 74 years, and 59.4% patients were male. Median Charlson Comorbidity Index (CCI) was 2 (0–2) with hypertension (27.2%), chronic kidney disease (27.2%), and cardiac comorbidities (22.7%) being the most frequent. Primary BSI was present in 17.7% of episodes, secondary bacteraemia including urosepsis in 31.3%, pneumonia in 31%, and influenza in 20%. Median SOFA score at the start of an episode was 2 (2–4). Septic shock was present in 1% of episodes and bacteraemia in 53.9%. Median LOS was 7 (4–13) days, and there was an ICU admission rate after suspected sepsis of 17.7% with a median ICU LOS of 4 (2–9) days. In-hospital mortality was 10.8%. Therefore, 96 (23.6%) episodes were classified in the worse outcome group and 310 (76.4%) episodes in the less severe outcome group.

**Table 1:** Patient demographics and characteristics, diagnosis of infection, disease severity and patient outcomes

	Total *n*=406 episodes, 397 patients
Number of episodes^a^	
One episode	388 (97.7)
Two episodes	8 (2.0)
Three episodes	1 (0.3)
Demographics^a^	
Age (years, median (IQR))	74 (64–74)
Sex (male)	236 (59.4)
Department of inclusion	
Emergency department	381 (93.8)
Infectious diseases	16 (3.9)
Haemodialysis	9 (2.2)
Comorbidities^a^	
CCI (median (IQR)	2 (0–2)
Cardiac	90 (22.7)
Hypertension	108 (27.2)
Cerebrovascular disease	48 (12.1)
Chronic pulmonary disease	64 (16.1)
Chronic kidney disease	108 (27.2)
Liver disease	17 (4.3)
Dementia	28 (7.1)
Diabetes	76 (19.1)
Solid malignancies	83 (20.9)
Haematological malignancies	15 (3.8)
Diagnosis of infection	
Primary bacteraemia	72 (17.7)
Secondary bacteraemia	127 (31.3)
Bacterial pneumonia	126 (31.0)
Influenza	81 (20.0)
Disease severity*	
SOFA (median, IQR))	2 (2–4)
SOFA (1)	73 (18.0)
SOFA (≧2)	333 (82.0)
Septic Shock	4 (1.0)
Bacteraemia	217 (53.4)
Outcome	
Length of stay (days, median (IQR))	7 (4–13)
ICU admission	72 (17.7)
ICU length of stay (days, median (IQR))	4 (2–9)
Mortality	44 (10.8)
Worse outcome^#^	96 (23.6)

^a^Demographics, except department of inclusion, and comorbidities are shown on patient level, not on episode level. *Disease severity based on SOFA score at the start of a new episode. ^#^Patients who were admitted to the ICU or who died during hospitalization were classified in the worse outcome group. All variables are presented as number (%) unless otherwise specified

### Supervised differential expression analyses

**Fig. 1:**
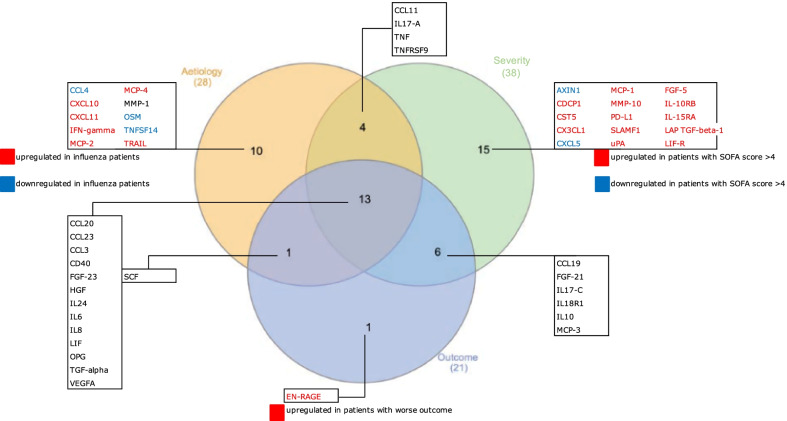
Venn diagram showing all differentially expressed proteins between three different groupings, together with overlapping findings. Three different groupings are: aetiology (influenza versus bacterial), severity (SOFA score < 2 versus > 4), and outcome (less severe outcome versus worse outcome)

Figure 1 shows a Venn diagram of all proteins that were significantly differentially expressed for three different groupings. In total, 28/92 proteins were significantly differently expressed when comparing patients with influenza (with or without pneumonia) with patients with bacterial infections. All but six of these proteins (CCL11, CXCL11, IFNγ, MCP-2, SCF, and TRAIL) were significantly less expressed in patients with influenza. Thirty-eight proteins were significantly differently expressed comparing patients with high SOFA score (> 4) and patients with low SOFA score (< 2). All but two proteins (AXIN1 and CXCL5) were significantly more expressed in patients with high SOFA score (> 4). In addition, 21/92 proteins were significantly different in patients with worse outcome compared to patients with less severe outcome. All proteins, except SCF, were significantly more expressed in patients with worse outcome. Nineteen of these proteins were also differentially expressed comparing patients with high and low SOFA score. Mean expression and *p* values for the 3 groupings are shown in Additional file 1: Tables S2, S3, and S4, respectively.

### Unsupervised clustering

#### Principal component analysis

The first four main principal components explained 42% of the variance in the data set (Additional file 1: Table S1, Fig.S2 and Fig. S3). Therefore, the following analyses were performed based on plotting these four components with each other. Gender, age, or batch confounding did not seem to be present in the normalized data set (Additional file 1: Fig. S1).

**Fig. 2:**
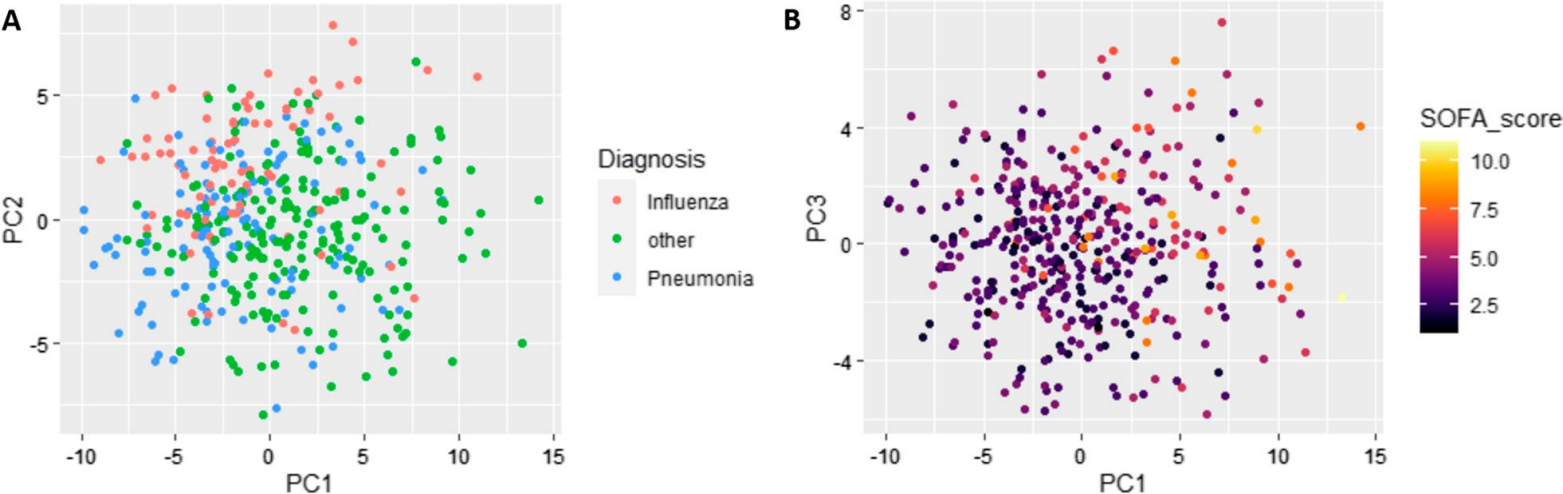
**A** principal component analysis to detect clustering between patients with influenza (pink), bacterial pneumonia (blue) and other bacterial infections (green). **B** principal component analysis to detect clustering based on SOFA score

Figure 2 shows the results of the principal component analyses. To look for distinct diagnosis-based clusters, inflammatory protein related profiles were initially checked by classifying patients in three diagnosis groups: influenza, bacterial pneumonia and other bacterial infection (Fig. 2A). Bacterial pneumonia and influenza separated most strongly from each other as well as from other bacterial infections based on the PC2 axis. This indicates that the infection site or aetiology of infection can at least to some extent determine the inflammation associated protein expression in response to an infection. However, separation clearly experienced some overlap between the different diagnosis groups and was not absolutely distinct.

Interestingly, SOFA score seemed to grasp and identify the differences in protein expression accurately (Fig. 2B). Patients with high SOFA scores clearly clustered together and differed from patients with low SOFA scores.

#### Hierarchical clustering

**Fig. 3:**
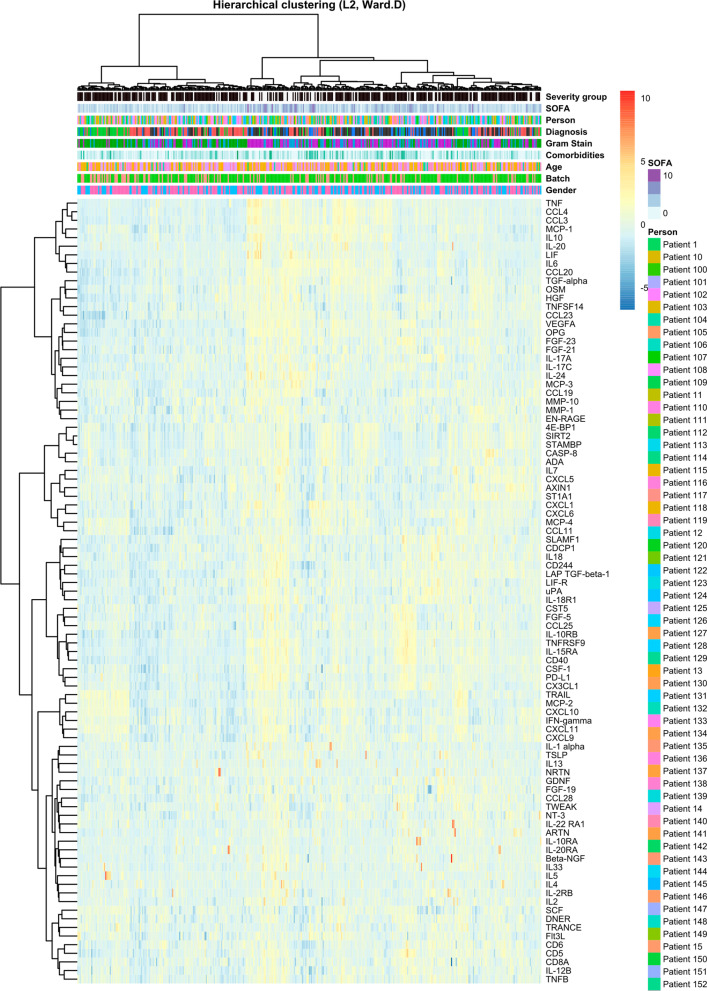
Hierarchical clustering plot

Results are shown in Fig. 3. Hierarchical clustering confirmed that no confounding was induced by sex, age, or batch in the normalized data set. Two distinct ‘Influenza’ groups could be distinguished at the extreme left and right of the cluster. The group on the left consisted mostly of patients with influenza without chest X-ray abnormalities, while the group on the right were mostly patients with influenza and chest X-ray abnormalities. Although at first it seemed that influenza clustered differently from bacterial pneumonia and other bacterial infections, thorough comparison of patients in different clusters revealed a pattern of severity that was considered as the underlying basis upon which the hierarchical clustering provided the presented dendrogram. In that same light, most patients with pneumonia and patients with other infections that clustered at the left of the dendrogram had lower SOFA scores than their variants situated at the right of the dendrogram. Furthermore, highest SOFA scores, and most BSIs (considered invasive disease) were seen in patients that clustered in the centre of the dendrogram. In addition, when comparing outcomes, those patients with worse outcome mostly clustered in the middle, while the left and right clusters on the dendrogram mostly contained patients with less severe outcome. Therefore, hierarchical clustering could confirm supervised differential expression, i.e., that some differences between viral (influenza) and bacterial infections were found, but that inflammatory proteins more strongly indicated disease severity.


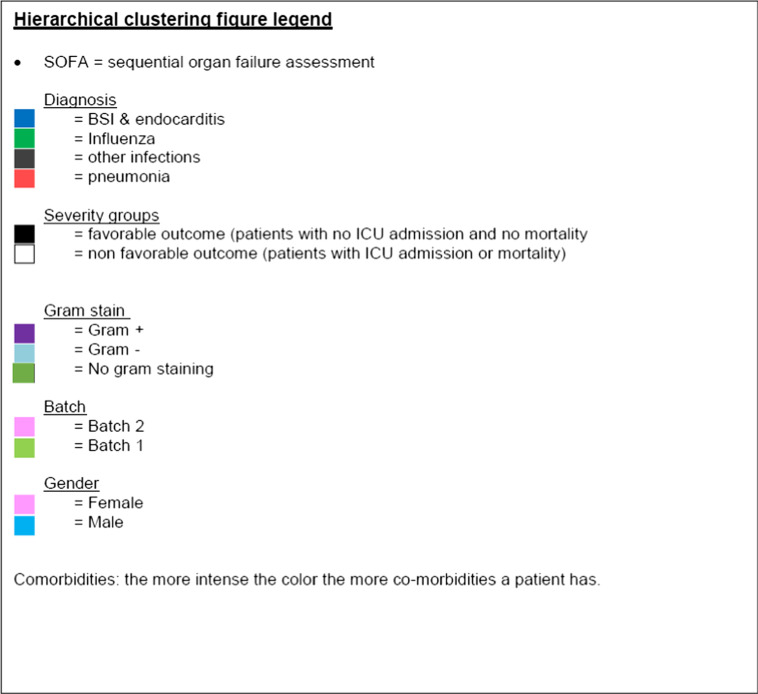

### Pathway enrichment analysis

**Fig. 4:**
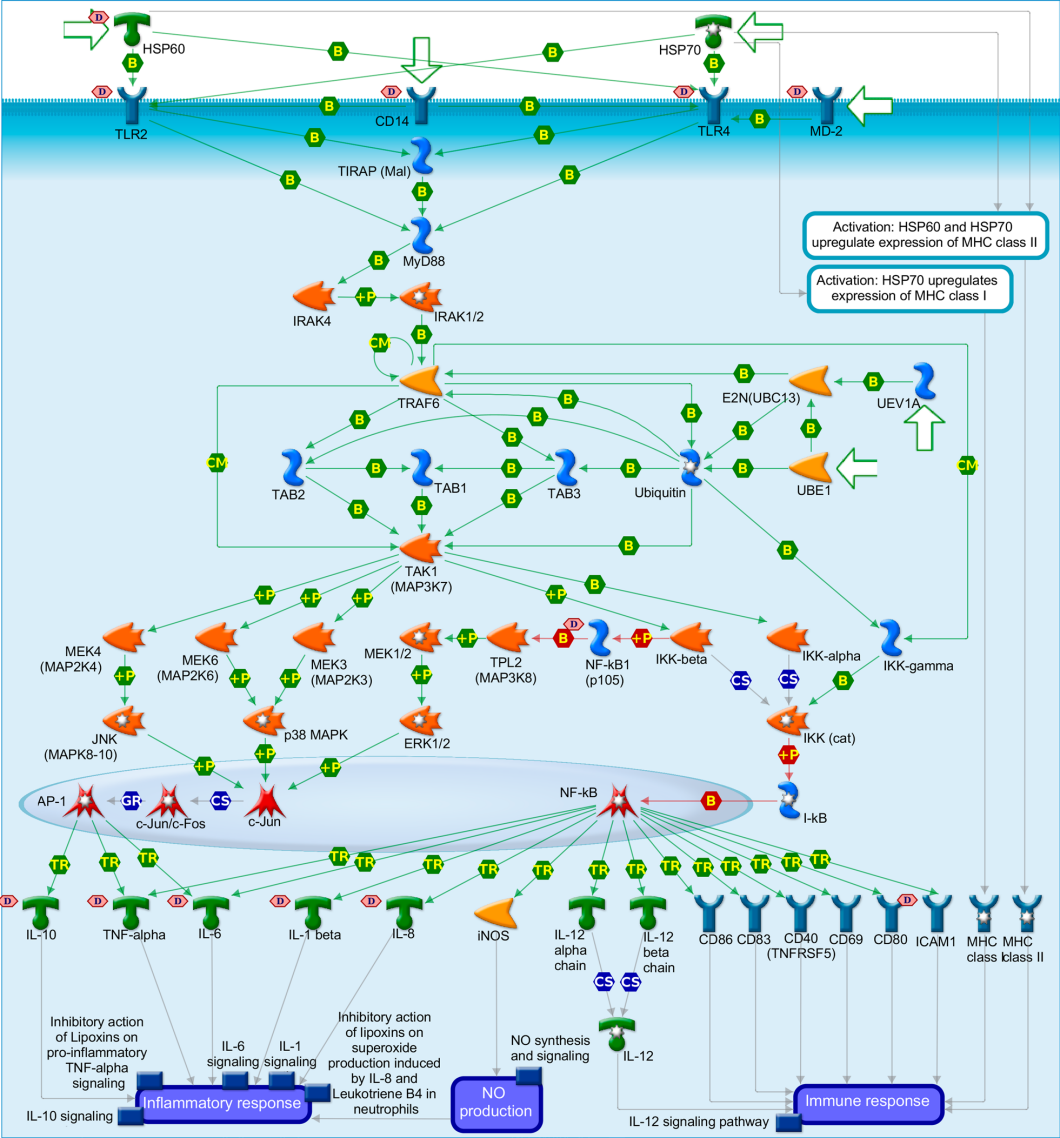
HSP60 and HSP70/TLR signalling pathway involved in the activation of the inflammatory response

Pathway enrichment analysis was done to illustrate the molecular mechanisms involved in this population with sepsis as the inflammatory panel used has been compiled based on knowledge from a range of inflammatory diseases [23]. The most important pathway involving proteins differentially expressed between patients with influenza and patients with bacterial infections was HMGB1/TLR signalling pathway. In proteins differentially expressed between patients with worse and less severe outcome, ERBB family/HGF signalling and chemotaxis/CCR1 signalling were considered as the two most important pathways involved. These pathways show shared entities and provide a clear link with IL-10 signalling, IL-1 signalling, and HSP60 and HSP70/TLR signalling pathways, known to be involved in sepsis. In the latter three pathways, proteins were downregulated in patients with influenza and upregulated in patients with worse outcome, illustrating differences in disease severity. Figure 4 shows up- and downregulated proteins in the HSP60 and HSP70/TLR signalling pathway.

### Prediction biomarkers

**Fig. 5:**
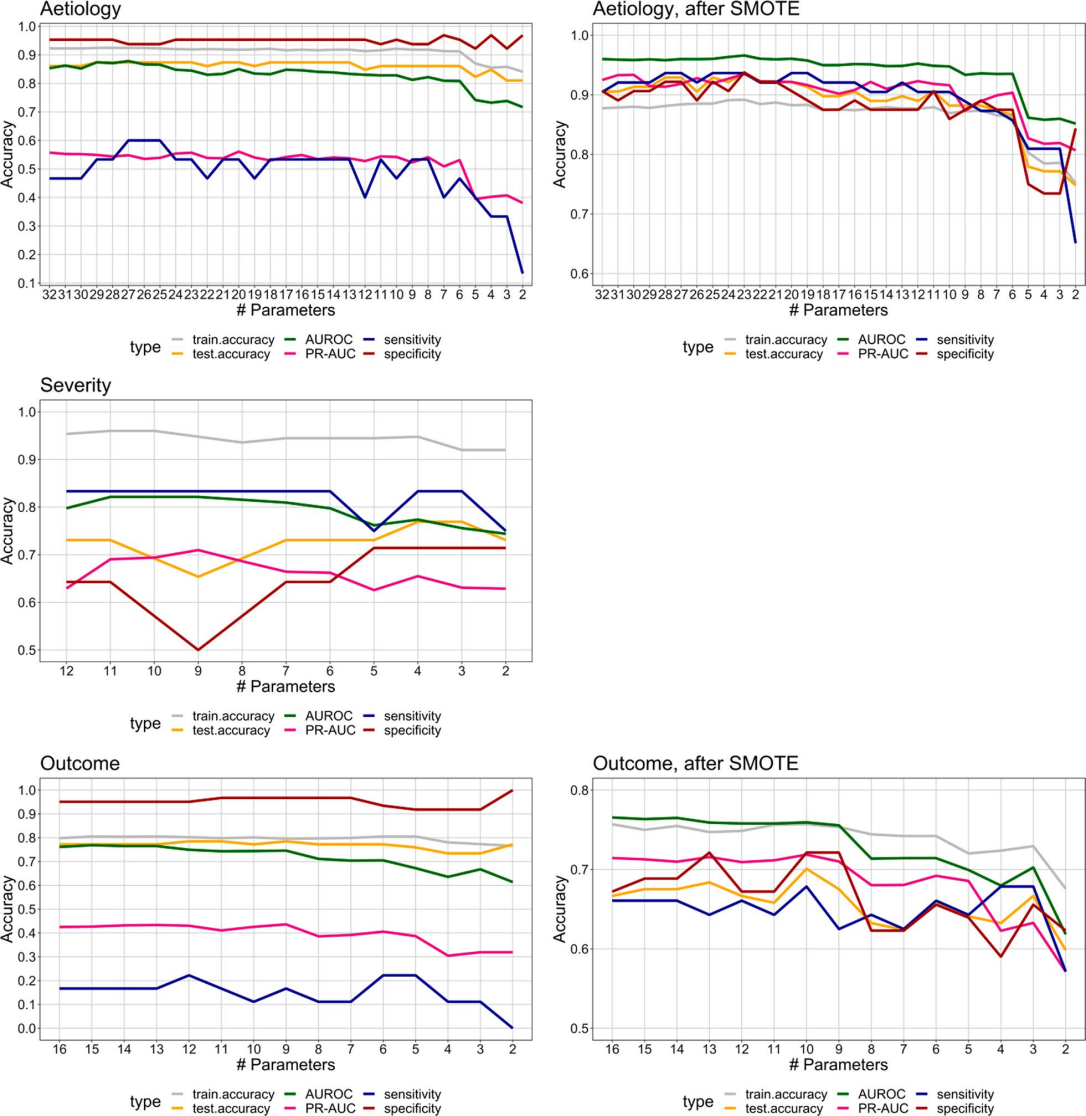
Model optimization for aetiology, disease severity and outcome, starting from the maximum number of parameters resulting from the elastic net regression models. The most optimal model was chosen based on the minimum decline of 5% in area under the ROC curve (AUROC) and sensitivity with the lowest number of proteins in the model. Aetiology: viral vs. bacterial sepsis, the most optimal model had an AUROC of 94% and sensitivity of 86% with six proteins in the model. Disease severity: SOFA score > 4 vs. SOFA score < 2, the most optimal model had an AUROC of 80% and a sensitivity of 83% with six proteins in the model. Outcome: worse vs. less severe outcome, the most optimal model had an AUROC of 76% and a sensitivity of 68% with ten proteins in the model. The most optimal models for aetiology and outcome were chosen after SMOTE procedure. AUROC, PR-AUC, sensitivity and specificity are shown

**Fig. 6:**
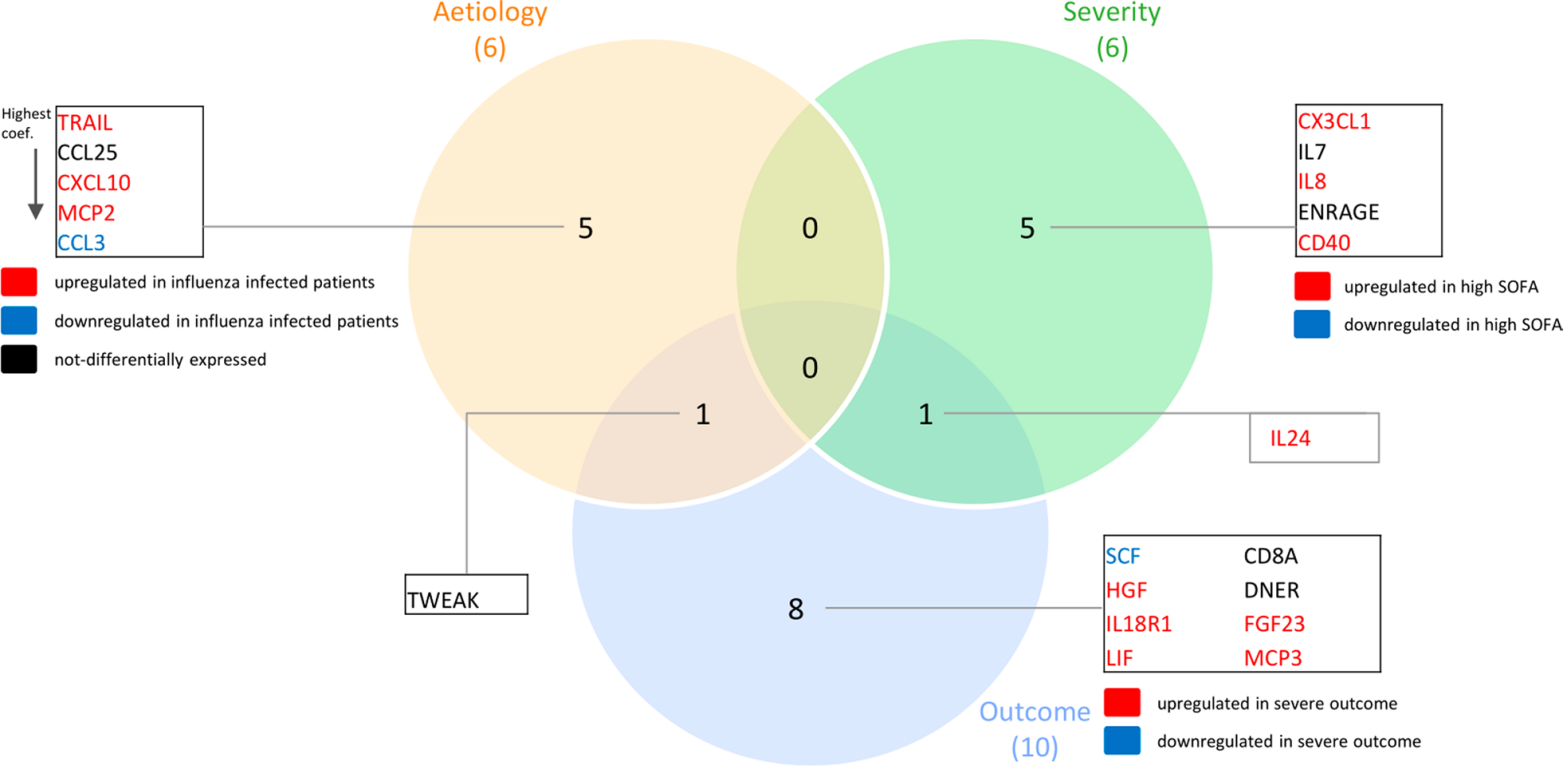
Proteins in the most optimal models, accurately predicting differences in aetiology, disease severity and outcome

The accuracy (area under the receiver-operating curve (AUROC) of all elastic net models was tracked from the maximum number of biomarkers using a stepwise backward selection. Due to the imbalance classes (minority and majority) in our data in aetiology and outcome models (79 viral vs. 322 bacterial episodes in the aetiology model and 94 episodes with worse outcome vs. 307 episodes with less severe outcome), Synthetic Minority Oversampling Technique (SMOTE) was implemented to produce a more balanced data set. This technique works by selecting the nearest neighbours of minority data points and generating new synthetic samples along the line between the minority sample and its neighbours [24]. After applying this technique, 188 and 237 new synthetic samples were generated in the worse outcome class of the outcome model and in the viral (influenza) category of the aetiology model, respectively. Model performances before and after SMOTE are shown in Fig. 5, and 5% decline in AUROC or sensitivity has been chosen to indicate a cutoff point. Proteins in the most optimal model are shown in Fig. 6. Six proteins could predict viral vs. bacterial infection with an AUROC of 94%, and a sensitivity and specificity of 86% and 87%, respectively. Six proteins could predict SOFA score > 4 vs. SOFA score < 2 with an AUROC of 80%, a sensitivity of 83% and a specificity of 65%. Ten proteins could predict less severe outcomes from worse outcome with an accuracy of 76%, a sensitivity of 68% and a specificity of 72%. In addition, the precision–recall AUC (PR-AUC), which is less prone to imbalanced data, was 91, 66, and 72% for the aetiology, severity, and outcome models, respectively. Comparatively, AUROC and specificity of these models was higher, while sensitivity was lower, than AUROC of routine biomarkers CRP, WBC and serum lactate separately in this cohort. Additional file 1: Fig. S4 shows ROC to predict aetiology (AUROC: 75.5, 73.4 and 66.5%, respectively), outcome (AUROC: 67.7, 58.5 and 69.7%, respectively) and severity (AUROC: 61.8, 52.0 and 69.1%, respectively) together with sensitivity and specificity.

## Discussion

In this study, we identified plasma proteins as potential biomarkers for early aetiologic diagnosis and prognosis of patients with suspected sepsis. One-third of a set 92 plasma proteins of inflammation were differentially expressed in viral infections (influenza) compared to infections of bacterial origin. Moreover, patients with severe disease (high SOFA score) and worse outcome (mortality or ICU admission) had significantly higher protein concentrations compared to patients with less severe disease and less severe outcome.

Not surprisingly, most identified proteins of the worse outcome group overlapped with those in patients with severe disease (high SOFA score), providing further proof that disease severity and patient outcome are related and associated with the inflammatory response to sepsis. Differences in protein expression related to inflammatory (IL-1), anti-inflammatory (IL10) and HSP pathways are associated with worse outcome and a model using combinations of quantitative protein expression levels could moderately discriminate worse from less severe outcome, as measured by ICU admission or mortality, among a non-severely ill cohort of patients with sepsis from selected sources. Three models consisting of several protein biomarkers were built. The aetiology model is particularly helpful for decisions whether to start empirical antibiotics in patients with suspected sepsis, the other two can point to the need for more intensive monitoring. Furthermore, SOFA score correlates well with this comprehensive analysis in the severity model and shows the usefulness of a cost-free scoring system.

Extensive research has previously been performed to search for accurate and sensitive biomarkers for sepsis [25–27]. Most biomarkers have their limitations, especially in differentiating between sepsis and other inflammatory syndromes [25, 28]. Similar to our findings, plasma biomarkers are often shown to be more strongly related to disease severity rather than to the microbial aetiology of infection, and the majority of biomarkers have been evaluated for their use in the prognosis of patients with sepsis [25–27]. When comparing these inflammatory markers to routine clinical and laboratory markers, that are already widely used by clinicians, these biomarkers show a similar accuracy of approximately 80% for the prognosis [15]. Moreover, when combining routine parameters with inflammatory biomarkers, similar performances were found (data not shown).

The availability of a commercial 92-plex panel for the detection of inflammation proteins allowed us to identify up to 38 differentially expressed proteins in this suspected sepsis population. Our results are in line with previous studies showing that a combination of several proteins more accurately differentiate between various disease severity profiles in sepsis and other infections, compared to single biomarkers [25, 29, 30]. However, our research, combining multiple biomarkers led to optimal models with as few biomarkers as possible for future applications in clinical practice. Furthermore, a proteomics approach has been used in other studies showing beneficial results as well [31]. In addition, combining proteomics with metabolomics, could provide an even clearer picture of host response in sepsis and provide answers to the current diagnostic and prognostic challenges [32].

Pathway enrichment analysis illustrated involvement of several pathways of early inflammatory response with several links to other pathways known to be involved in sepsis. In differentially expressed proteins, pathway enrichment analysis confirmed the involvement of ERBB family/HGF signalling and chemotaxis/CCR1 signalling pathways for less severe or worse outcome and HMGB1/TLR signalling pathway for aetiology. The ERBB family is a family of tyrosine kinases known to be involved in cancers, because they stimulate cell proliferation but are also known to play a role in activation and recruitment of inflammatory cells. The chemotaxis/CCR1 signalling pathway is involved in chemokine production and neutrophil recruitment, and the HMGB1/TLR signalling pathway is a well-known pathway of Toll-like receptors that induce inflammation and the early immune response [33]. Not surprisingly, these pathways have similarities and shared entities with other pathways known to be involved in sepsis. For example, the IL-10 signalling pathway is involved in the inhibition of the immune response, and the IL-1 and HSP60 and HSP70/TLR signalling pathways are involved in activation of the innate and adaptive immune response. The observation was made that proteins in both inflammatory activation and inhibition are upregulated in patients with worse outcome, which is proof of the dysregulated immune response. An increase in early proteins and especially chemokines is also reported by others [34–37], suggesting that higher levels of chemokines are necessary because of reduced neutrophil migration velocity, eventually leading to an impaired immune response which is associated with disease severity and lower survival rates [35–37]. The identified pathways have also been shown to be related to disease severity in COVID-19 patients [34].

Indeed, the observation that inflammatory protein expression profile is related to sepsis severity, has recently been described in patients with COVID-19, using the same targeted proteomic approach [38]. Differentially expressed proteins in COVID-19 patients were involved in the same pathways found in the present study [29, 34, 38]. This strengthens our finding that these proteins could be more useful for the prognosis of patients with severe infections, rather than for the aetiologic diagnosis.

One major strength of this study is the use of a large cohort of patients who were prospectively included and uniformly defined. The results of this study show the importance of the host response in predicting patient outcome. The unicentric nature of this study and the lack of comparison to physician judgement and standard laboratory studies is a limitation resulting in a difficult extrapolation to other centres. Furthermore, we did not compare our findings with a non-sepsis control population and, therefore, lack information on basic expression levels. In addition, all patients included in this study were selected based on the presence of having a type of infection. Of course, this creates some selection bias, and leaves out many other diagnoses that make up a significant proportion of patients with sepsis, such as intra-abdominal infection without bacteraemia, skin/soft tissue infection without bacteraemia, CNS infection, other viral infections. Together with not including a large number of patients with suspected sepsis at the ED that end up having negative cultures or a non-infectious diagnosis, this creates a selection bias when attempting to generalize these results to clinical care, where undifferentiated patients are tested in the ED setting. Last, there were no follow-up plasma samples during patients’ hospitalization, and we were not able to assess the impact of later events on patient outcome.

In conclusion, the inflammatory response of patients with viral infection differed from the response of those with bacterial infection. However, the inflammatory response correlated more strongly with disease severity and worse patient outcome. An increase in these proteins was seen for patients with worse outcome. Several proteins could accurately predict influenza, disease severity, or worse outcome. Although the diagnostic potential of these biomarkers for distinguishing bacterial from viral infections is limited, their value for prognosis can be high. This study confirms that sepsis is a complex syndrome with diverse backgrounds. Various cytokine networks are activated which can lead to the uncontrollable inflammatory response that results in a uniform sepsis condition. Therefore, the search for accurate biomarkers remains difficult. Comprehensive cytokine analyses such as these can help in gaining further insight but are presently not suitable for direct use in the clinic. However, in the future, the differentially expressed proteins identified in this study could be incorporated in a scoring algorithm or low-cost point of care test. Routine use of such an algorithm or test at the ED could help in early outcome prediction, guide close monitoring, and direct diagnostics, to help diagnose fatal disease in an early stage, ultimately leading to better management of patients with suspected sepsis.

## Supplementary Information

**Additional file 1.** Additional methods, figures and tables.

## Data Availability

The data sets used and analysed during the current study are available from the corresponding author on reasonable request. The data are not publicly available due to them containing information that could compromise research participant privacy/consent.
