# Supplementary material for: A prospective observational cohort study to identify inflammatory biomarkers for the diagnosis and prognosis of patients with sepsis
Source: J Intensive Care. 2022 Mar 9;10:13. doi: 10.1186/s40560-022-00602-x (PMC8905560; doi:10.1186/s40560-022-00602-x)
Supplement: Supplementary file 1 — Additional file 1. Additional methods, figures and tables. [file 40560_2022_602_MOESM1_ESM.docx]

**Additional Files**

**Additional Methods**

*Supervised differential expression analyses*

Differential expression analyses were performed with a Welch’s t test also known as the unequal variances t-test. Differences were searched for all 92 proteins across patients with influenza (with and without pneumonia) and patients with other bacterial infections. In addition, differences were analysed according to severity (high SOFA score >4 versus low SOFA score <2) and to outcome (worse versus less severe outcome). Pairwise comparisons for each protein were made according to Petrera *et al.* (1). The basic ‘stats’ R-package was used. A Bonferroni multiple testing correction was performed. A p-value < 0.05 was considered significantly differentially expressed.

*Unsupervised clustering*

Initial data exploration and data dimensionality reduction was done using principal component analysis (PCA). PCA is well known to highlight the most important aspects of data variability and de-emphasizes the others. The R-packages ‘ggplot2’, ‘ggreppel’ and ‘GGally’ were used to reveal the internal data structure by using an eigenvector-based multivariate approach.

Following supervised analysis, Hierarchical clustering, a commonly used unsupervised analysis method to check similarity between subjects, was used. In this analysis, samples that grouped together were considered to be more similar than samples from other patients in the cohort, i.e., they have comparable inflammatory protein patterns. Hierarchical clustering was performed using a Euclidean distance calculation method in combination with a Ward.D clustering method. R-packages ‘dplyr’, ,’randomcoloR’ and ‘ggplot2’ were loaded to complete the clustering. Clustering and distance calculation was performed by Ward.D clustering using Euclidean distance due to its ability to capture larger variations within the data avoiding splintered clustering. As the method is very sensitive to outliers, the control samples were excluded from the analysis.

*Pathway enrichment analysis*

Pathway enrichment analyses were performed on significantly differentially expressed proteins in MetaCoreTM. Protein Uniprot ID’s were searched in the light of their molecular function as well as their biological process. These analyses help gain mechanistic insight into gene lists and identify biological pathways that are enriched in a protein list, more enriched than would be expected by chance. Afterwards, proteins shared between these pathways and other pathways previously known to be involved in sepsis were integrated in the latter to allow for a better understanding of the role of these inflammatory biomarkers.

*Elastic Net*

To search for the best biomarker predictors of different outcomes, elastic net regression was selected as an alternative regularization technique that combines the L1 and L2-penalizations. This method does automatic variable selection and continuous shrinkage and produces a sparse model with better prediction accuracy, particularly in the two-class classification method (2).

Three categorical variables: aetiology (viral vs. bacterial infection) disease severity (based on SOFA score) and outcome (worse vs less severe) were proposed as vector of dependent variable $\boldsymbol{y}$ in three different models. The tuning parameter $\lambda$ controls the penalty factor and was chosen by 3-repeated 5-fold cross-validation. The models were trained in 80% of the data and the remaining was used for calculating the prediction accuracy and the area under the curve (AUC). All proteins were inserted in the starting model. The most optimal model was chosen based on the AUC. All estimations were performed in R *glmnet* and *ROCR* packages (3, 4).

**Additional Figures**


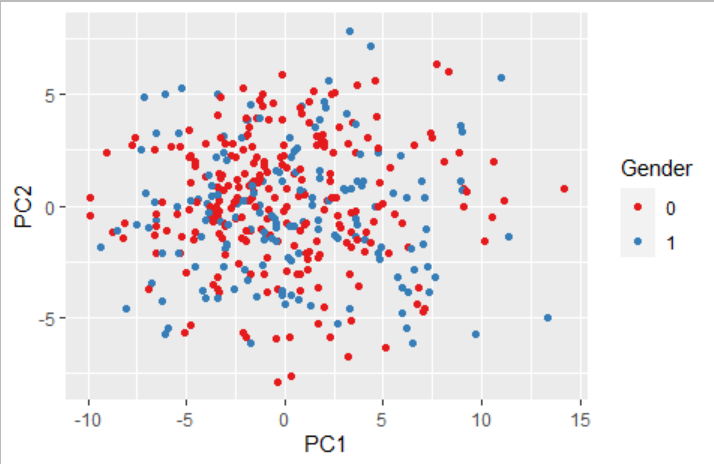


**a**


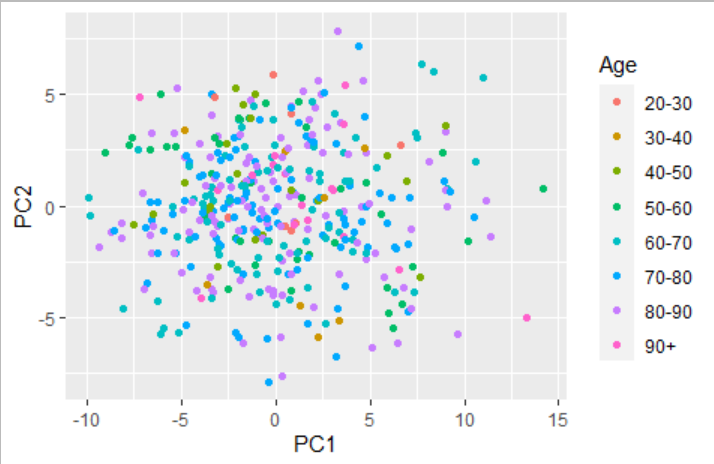


**b**

**c**


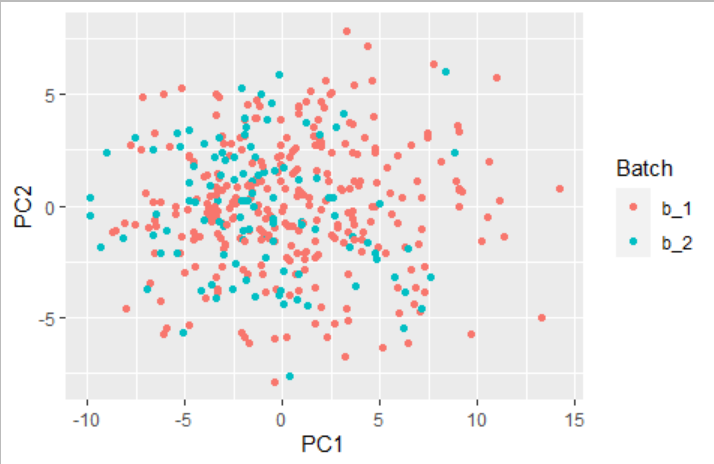


**Additional Figure 1**. Principal component analysis with groups based on gender (a), age (b), and batches (c). These were identified as not confounding.


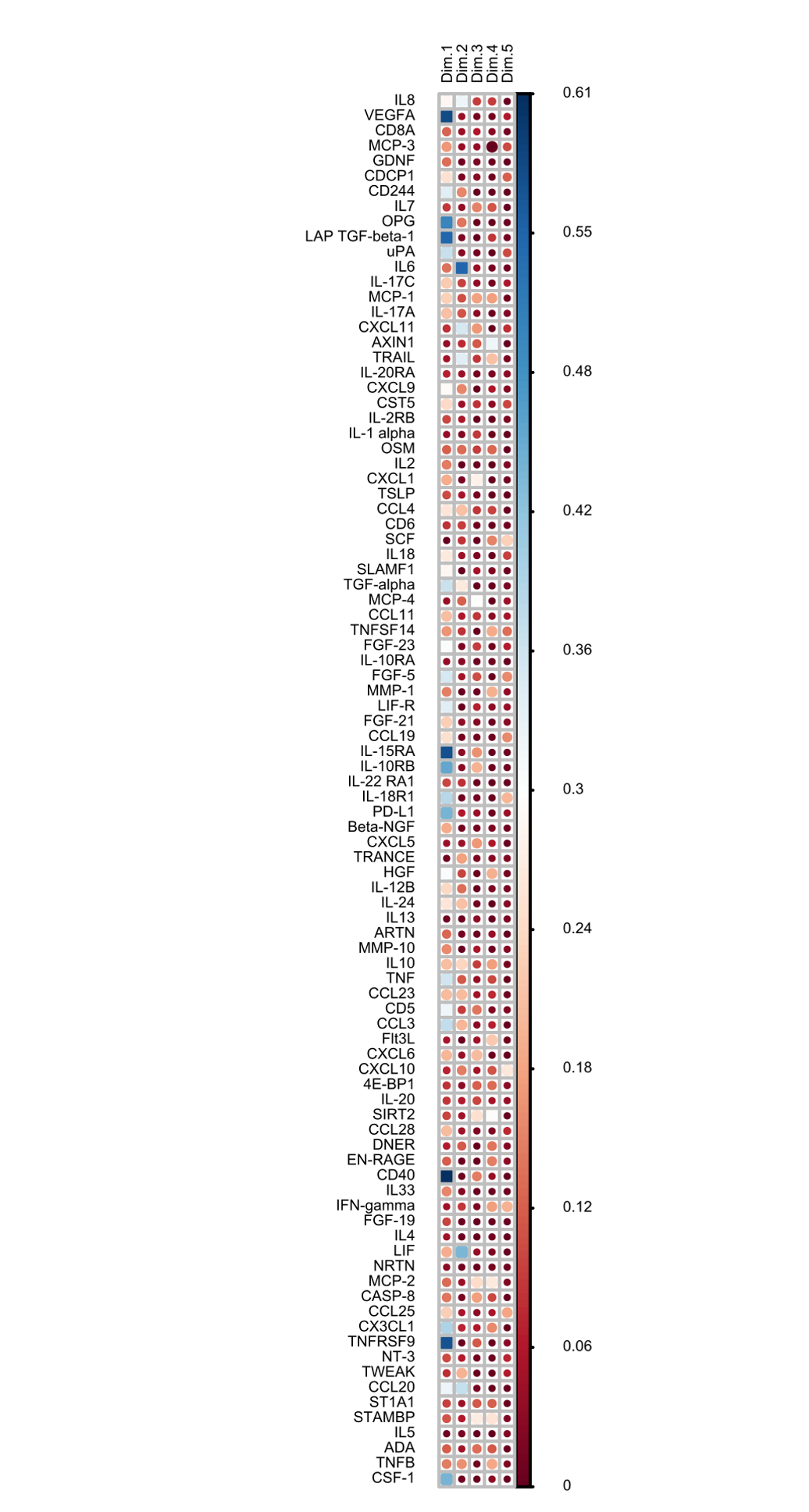


**Additional Figure 2.** The squared coordinates(cos2) plot shows the quality of representation of the proteins on a factor map for the first five dimensions. A high cos2 indicates a good representation of the protein on the corresponding principal component.


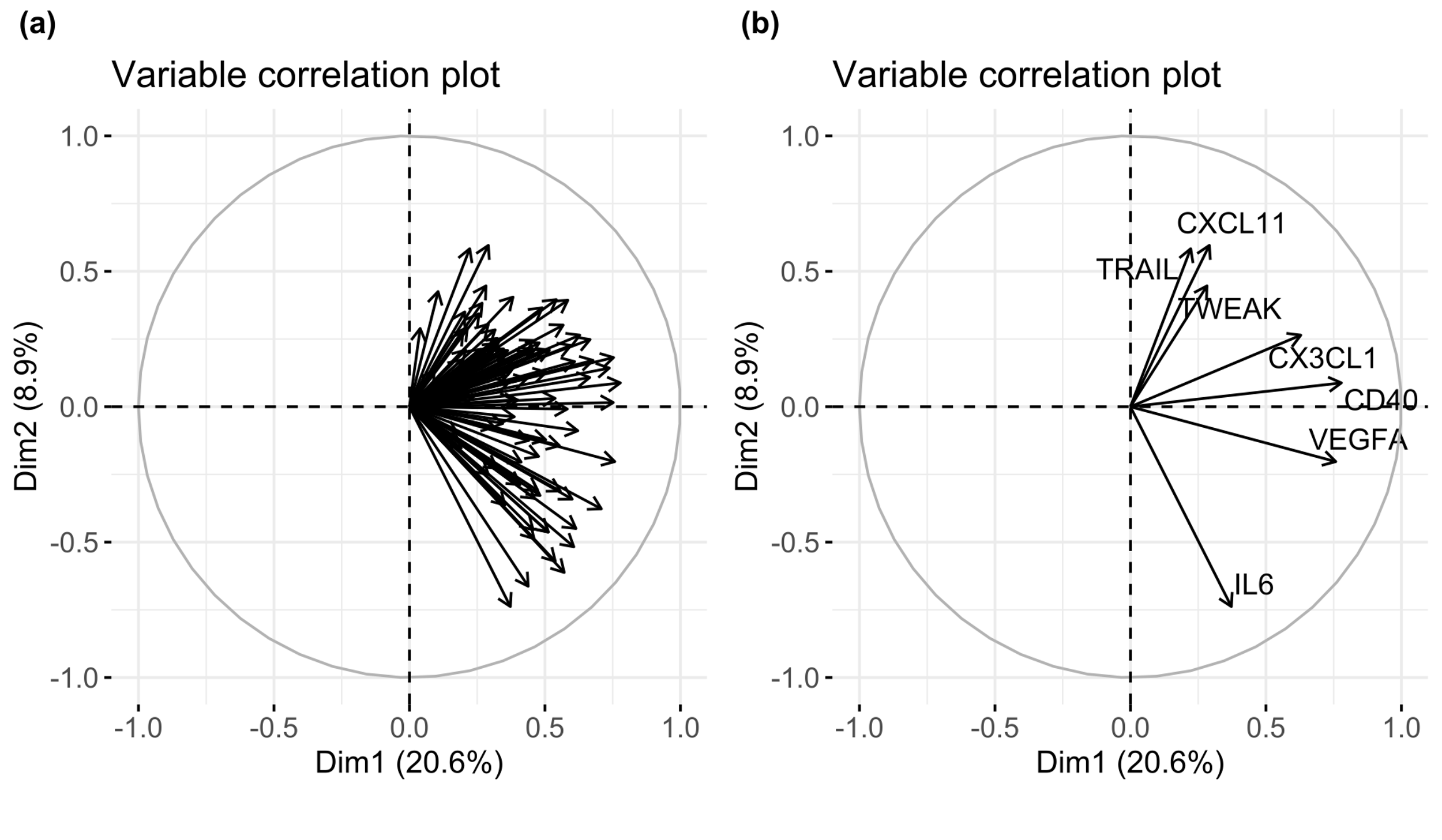


**Additional Figure 3.** Variable correlation plot that allows visualizing how proteins relate to one another and at the same time indicates how each protein contributes to each principal component. A) All 92 proteins are shown. B) Subset of variables are shown, representing proteins with extreme coordinates values on either PC1 or PC2.

**Additional Figure 4.** Receiver operating curves and performance characteristics of routine biomarkers separately for this study cohort. AUROC: area under the receiver operating curve; Sens.: sensitivity; Spec.: specificity.

**Additional Tables**

**Additional Table 1.** The first five main principal components (PC). The first four PC explained 42% of observed variance.

|  | **PC1** | **PC2** | **PC3** | **PC4** | **PC5** |
| --- | --- | --- | --- | --- | --- |
| Standard deviation | 4.3485 | 2.8538 | 2.4206 | 2.3905 | 1.8831 |
| Proportion of variance | 0.2055 | 0.0885 | 0.0637 | 0.0621 | 0.0386 |
| Cumulative proportion | 0.2055 | 0.2941 | 0.3578 | 0.4199 | 0.4584 |

**Additional Table 2.** Differentially expressed proteins between patients with influenza and patients with bacterial infections.

| **Protein** | **Influenza (n = 79)** | **Bacterial infections (n = 322)** | | **p-value** |
| --- | --- | --- | --- | --- |
| IL8 | 7.78536778481013 | | 8.5816851242236 | 0,000278 |
| CCL11 | 7.56094911392405 | | 7.20074456521739 | 3,98E-06 |
| CCL20 | 9.05340234177215 | | 10.5492861490683 | 3,00E-10 |
| CCL23 | 11.3601117721519 | | 12.0562967701863 | 9,43E-12 |
| CCL3 | 6.25293132911392 | | 7.49321649068323 | 4,34E-15 |
| CCL4 | 7.38342588607595 | | 8.48298155279503 | 3,45E-11 |
| CD40 | 12.5934606329114 | | 12.9362190993789 | 0,000358 |
| CXCL10 | 13.1371136075949 | | 11.7446202484472 | 1,90E-20 |
| CXCL11 | 11.73139 | | 10.2591934161491 | 5,45E-14 |
| FGF-23 | 4.46214069620253 | | 5.30152024844721 | 0,000442 |
| HGF | 10.4572824050633 | | 11.1182611490683 | 3,34E-07 |
| IFN-gamma | 10.5822778481013 | | 9.48106248447205 | 8,78E-05 |
| IL-17A | 2.94531563291139 | | 4.18545459627329 | 2,36E-11 |
| IL-24 | 2.24880151898734 | | 2.85692711180124 | 3,76E-06 |
| IL6 | 8.36291056962025 | | 10.0216559006211 | 6,97E-08 |
| LIF | 1.84252620253165 | | 3.12973860248447 | 4,59E-14 |
| MCP-2 | 11.808945443038 | | 10.2177758385093 | 8,99E-14 |
| MCP-4 | 14.4441601898734 | | 13.8177433850932 | 2,68E-05 |
| MMP-1 | 10.8696085443038 | | 11.4420218322981 | 0,000183 |
| OPG | 11.4179899367089 | | 11.7510125465839 | 0,000331 |
| OSM | 7.12268069620253 | | 7.94123782608696 | 1,90E-06 |
| SCF | 8.92490069620253 | | 8.53394726708074 | 0,000306 |
| TGF-alpha | 4.9039946835443 | | 5.88156913043478 | 3,80E-17 |
| TNF | 4.74096626582278 | | 5.51113894409938 | 5,58E-07 |
| TNFRSF9 | 7.34747436708861 | | 8.00897704968944 | 1,59E-06 |
| TNFSF14 | 5.73642911392405 | | 6.31825776397516 | 2,25E-08 |
| TRAIL | 8.1188232278481 | | 7.20759726708075 | 5,77E-15 |
| VEGFA | 6.80248393433742e-06 | | 11.7888084177215 | 6,80E-06 |

**Additional Table 3.** Differentially expressed proteins according to disease severity comparing patients with high SOFA score (>4) and patients with low SOFA score (<2).

| **Protein** | **SOFA score <2**  **n = 72** | **SOFA score >4**  **n = 62** | **p-value** |
| --- | --- | --- | --- |
| IL8 | 7.54511253424658 | 10.0506224193548 | 4.13823210801972e-13 |
| VEGFA | 11.9251419863014 | 12.6223388709677 | 1.49187362333609e-10 |
| MCP-3 | 3.38130623287671 | 4.45425661290323 | 3.66289033978312e-05 |
| CDCP1 | 4.51357815068493 | 4.98085096774194 | 0.000500836359188533 |
| OPG | 11.5039200684931 | 12.1871877419355 | 7.99483948196142e-09 |
| LAP TGF-beta-1 | 8.29018849315069 | 8.626335 | 0.000317647211401689 |
| uPA | 10.4486979452055 | 10.8321574193548 | 3.05162879708093e-05 |
| IL6 | 8.86928554794521 | 11.276035 | 1.39317027137116e-08 |
| IL-17C | 2.6196152739726 | 4.033145 | 2.6953516893144e-11 |
| MCP-1 | 13.2191141780822 | 14.1845708064516 | 1.00947687294954e-06 |
| IL-17A | 3.71904404109589 | 4.97488338709677 | 1.1639481020654e-05 |
| AXIN1 | 5.01794965753425 | 4.27246774193548 | 0.000735988023517331 |
| CST5 | 5.51110931506849 | 6.15005951612903 | 2.05813895879054e-05 |
| SLAMF1 | 2.16883678082192 | 2.61933064516129 | 4.1040070002183e-07 |
| TGF-alpha | 5.36495917808219 | 6.46573967741935 | 5.09686605947034e-09 |
| CCL11 | 7.10763821917808 | 7.43872774193548 | 0.000922599826720678 |
| FGF-23 | 4.19015582191781 | 7.05758241935484 | 7.75226544550476e-13 |
| FGF-5 | 1.52156767123288 | 1.80379822580645 | 2.80448223862097e-05 |
| LIF-R | 4.63568376712329 | 5.0726135483871 | 5.0757264192724e-07 |
| FGF-21 | 7.89396767123288 | 9.48193983870968 | 1.08627697613365e-05 |
| CCL19 | 10.7040767123288 | 11.7283843548387 | 9.12166070276976e-09 |
| IL-15RA | 1.96988150684932 | 2.79340741935484 | 6.43547608294061e-12 |
| IL-10RB | 6.44204938356164 | 6.8326964516129 | 1.09463172158329e-07 |
| IL-18R1 | 9.43435 | 9.98374274193548 | 1.47643179219557e-05 |
| PD-L1 | 7.83681493150685 | 8.63897822580645 | 4.19943850438217e-08 |
| CXCL5 | 11.300358630137 | 10.2860420967742 | 0.000125365074803325 |
| HGF | 10.849035890411 | 11.5256979032258 | 9.81866527526374e-05 |
| IL-24 | 2.2605798630137 | 3.85442806451613 | 1.69183896808857e-12 |
| MMP-10 | 9.26558061643836 | 10.0158332258065 | 2.44330240208854e-06 |
| IL10 | 7.55581910958904 | 9.54024225806452 | 5.42307099634175e-05 |
| TNF | 5.06843897260274 | 6.32501096774194 | 5.43325430470919e-05 |
| CCL23 | 11.8848928767123 | 12.3486670967742 | 4.04616129880677e-07 |
| CCL3 | 6.95585308219178 | 8.21465290322581 | 1.26654591461499e-05 |
| CD40 | 12.6932576712329 | 13.5788738709677 | 1.3824006239517e-11 |
| LIF | 2.40454287671233 | 4.36838129032258 | 3.03641088836534e-06 |
| CX3CL1 | 6.53158664383562 | 7.58355661290323 | 2.4191775014675e-13 |
| TNFRSF9 | 7.48065815068493 | 8.92591516129032 | 2.1438471275234e-09 |
| CCL20 | 9.63605212328767 | 11.3375485483871 | 1.24487781392583e-09 |

**Additional Table 4.** Differentially expressed proteins according to outcome comparing patients with worse outcome (in-hospital mortality and/or ICU admission) and patients with less severe outcome.

| **Protein** | **Less severe outcome (n = 307)** | **Severe outcome (n = 94)** | **p-value** |
| --- | --- | --- | --- |
| CCL19 | 10.9480729641694 | 11.4805571276596 | 1,92E-09 |
| CCL20 | 10.0244119543974 | 11.0063239893617 | 4,50E-07 |
| CCL23 | 11.8513747882736 | 12.1404715957447 | 0.000131 |
| CCL3 | 7.0633367752443 | 7.85481803191489 | 0.000216 |
| CD40 | 12.7870199022801 | 13.1354343617021 | 0.00043 |
| EN-RAGE | 3.94320885993485 | 4.41047367021277 | 0.000475 |
| FGF-21 | 8.31934986970684 | 9.19117138297872 | 0.0004255 |
| FGF-23 | 4.80573684039088 | 6.21529175531915 | 2,39E-08 |
| HGF | 10.85139 | 11.4343475531915 | 2,37E-08 |
| IL10 | 8.02952491856677 | 9.15281037234043 | 0.000536 |
| IL-17C | 2.96255576547231 | 3.53380026595745 | 9,85E-09 |
| IL-18R1 | 9.50566439739414 | 9.86210085106383 | 9,38E-09 |
| IL-24 | 2.53017377850163 | 3.41300531914894 | 0,00000791 |
| IL6 | 9.32308771986971 | 10.9090979255319 | 0,000000113 |
| IL8 | 8.10929723127036 | 9.45523845744681 | 0,000000261 |
| LIF | 2.5299790228013 | 4.00672170212766 | 1,79E-08 |
| MCP-3 | 3.56726726384365 | 4.33320771276596 | 2,37E-09 |
| OPG | 11.595607752443 | 11.978677287234 | 1,11E-09 |
| SCF | 8.73034951140065 | 8.22107313829787 | 2,39E-09 |
| TGF-alpha | 5.54186123778502 | 6.16946212765957 | 6,05E-08 |
| VEGFA | 11.9935900651466 | 12.3526069680851 | 1,79E-09 |

**Additional References**

1. Petrera A, von Toerne C, Behler J, Huth C, Thorand B, Hilgendorff A, et al. Multi-platforms approach for plasma proteomics: complementarity of Olink PEA technology to mass spectrometry-based protein profiling. bioRxiv. 2020:2020.08.04.236356.

2. Zou H, Hastie T. Regularization and variable selection via the elastic net.

3. Friedman J, Hastie T, Tibshirani R. Regularization Paths for Generalized Linear Models via Coordinate Descent. J Stat Softw. 2010;33(1):1-22.

4. Sing T, Sander O, Beerenwinkel N, Lengauer T. ROCR: visualizing classifier performance in R. Bioinformatics. 2005;21(20):3940-1.
